# Association of early life adversity with cardiovascular disease and its potential mechanisms: a narrative review

**DOI:** 10.3389/fpubh.2024.1341266

**Published:** 2024-01-24

**Authors:** Huiying Tan, Huiting Zhou, Jingmei Chen, Huixia Ren, Yi Guo, Xin Jiang

**Affiliations:** ^1^Department of Geriatrics, The Second Clinical Medical College, Jinan University (Shenzhen People’s Hospital), Shenzhen, China; ^2^The First Affiliated Hospital, Jinan University, Guangzhou, China; ^3^Department of Neurology, The Second Clinical Medical College, Jinan University (Shenzhen People’s Hospital), Shenzhen, China; ^4^Shenzhen Clinical Research Center for Geriatrics, Shenzhen People’s Hospital, Shenzhen, China

**Keywords:** early life adversity, adverse childhood experiences, cardiovascular disease, risk behavior, mental health

## Abstract

Strong epidemiological evidence has shown that early life adversity (ELA) has a profound negative impact on health in adulthood, including an increased risk of cardiovascular disease, the leading cause of death worldwide. Here, we review cohort studies on the effects of ELA on cardiovascular outcomes and the possible underlying mechanisms. In addition, we summarize relevant studies in rodent models of ELA. This review reveals that the prevalence of ELA varies between regions, time periods, and sexes. ELA increases cardiovascular health risk behaviors, susceptibility to mental illnesses, and neuroendocrine and immune system dysfunction in humans. Rodent models of ELA have been developed and show similar cardiovascular outcomes to those in humans but cannot fully replicate all ELA subtypes. Therefore, combining cohort and rodent studies to further investigate the mechanisms underlying the association between ELA and cardiovascular diseases may be a feasible future research strategy.

## 1 Introduction

Since Felitti et al. conducted the Adverse Childhood Experiences Study in 1998, the relationship between Early Life Adversity (ELA) and negative long-term health outcomes has attracted much research attention (1). Also called adverse childhood experiences in human studies, ELA is defined as potentially traumatic events that occur before adulthood, including threats to the child’s bodily, familial, or social safety and security, and ranges from broad categories of abuse, neglect, and household dysfunction to more specific experiences of bullying, exposure to crime, victimization, and economic hardship (1–4). ELA is highly prevalent, with regional variations occur. According to data from the Behavioral Risk Factor Surveillance System, 61.55% of adults in the United States have experienced ELA (2); in European countries, this figure ranges from 20.4 to 69.4% (5). In Chinese middle-aged or older adults it can be up to 80.9%, according to data from the China Health and Retirement Longitudinal Study (6). Societal changes over time affect the prevalence of specific adverse experiences. For example, during the first 15–18 years of the 21st century, parental illness, sibling death, exposure to domestic violence, childhood poverty, parental divorce, serious childhood illness, physical abuse, sexual abuse, physical and emotional bullying, and exposure to community violence declined; however, parental alcohol and drug abuse increased (7).

ELA exposure has been strongly associated with the development of various health conditions in later life, including cardiovascular disease, which is the leading cause of death worldwide. Cohort studies indicate that ELA increases the odds of cardiovascular health risk behaviors, such as smoking and alcoholism, as well as having long-term effects on the stress response system, leading to autonomic, vascular, and inflammatory dysfunction, and contributing to the onset and development of cardiovascular disease (4). Rodent models such as maternal separation have been developed to study the underlying mechanisms of cardiovascular effects of ELA. Consistent with human cohort studies, negative cardiovascular outcomes were observed (8).

This review provides an overview of the epidemiological evidence linking ELA to the development of cardiovascular disease and the mechanisms underlying the effects of ELA on the cardiovascular system. In addition, we summarize the rodent models that have been developed to mimic ELA and mechanistic studies performed using these models. Finally, we discuss the direction of future research.

## 2 ELA and cardiovascular outcomes in humans

An increasing number of cohort studies have shown ELA to be strongly associated with negative cardiovascular outcomes (Table 1). Different types of adversities have been carefully classified, and their effects studied. Abuse, neglect, and household dysfunction are the three most common types of adversity; all of these have been shown to increase the risk of cardiovascular events in adulthood, including ischemic heart disease, myocardial infarction, coronary heart disease, stroke, and heart failure (9, 11, 13–15, 18, 19, 21). These correlations were dose-dependent, the more types of adversities experienced, the higher the risk (9, 13, 18). Exposure to a single adversity type has been associated with a 1.3- to 1.7-fold increase in the risk of ischemic heart disease compared with that of individuals not exposed to adversity (9). The risk of developing myocardial infarction was increased in individuals who experienced ELA compared with those who did not, while the risk of developing coronary heart disease and stroke was increased in individuals exposed to more than four types of ELA compared with those who experienced fewer types (13). The risk of developing heart failure doubled following exposure to three to five types of ELA (18).

**Table 1:** Large cohort studies on the correlation of ELA with cardiovascular outcomes.

Type of adversity	Participants	Cardiovascular outcomes	References
Abuse (emotional, physical, sexual), Neglect (emotional, physical), Household dysfunction (substance abuse, mental illness, domestic violence, criminal household member, parental marital discord)	Health plan members at Kaiser Permanente’s Health Appraisal Center in San Diego from 1995 to 1997 *n* = 17,337, 54% female, 75% Caucasian, aged 56 ± 15.2 years	Risk of ischemic heart disease increased following exposure to any individual adversity except parental marital discord	Dong et al. (9)
Parental divorce, Financial difficulties, Interpersonal conflicts, Fear of a family member, Longstanding illness, Alcohol-related problems	Finnish citizens *n* = 23,916, 59% female, aged 20–24, 30–34, 40–44, 50–54 years	Women: risk of cardiovascular disease increased following exposure to financial difficulties, interpersonal conflicts and longstanding illness of a relative Men: increased risk following longstanding illness of a relative	Korkeila et al. (10)
Physical abuse, Sexual abuse	Nurses’ Health Study 2 participants *n* = 66,798, 100% female, aged 25–42 years	Physical and sexual abuse in childhood predicts early onset of cardiovascular events	Rich-Edwards et al. (11)
Witnessed violence with a weapon, Victims of violence	National Longitudinal Study of Adolescent Health participants *n* = 7,971, 55.4% female, aged 24–31 years	Increased risk of hypertension in males who witnessed violence and females who were victims of violence	Ford and Browning (12)
Physical abuse, Sexual abuse, Emotional abuse, Household member mental illness, Alcoholism, Drug abuse, Imprisonment, Divorce, Intimate partner violence	2010 Behavioral Risk Factor Surveillance System participants *n* = 53,998, 60.5% female, adults	Higher risk of myocardial infarction, coronary heart disease, and diabetes	Gilbert et al. (13)
Household dysfunction, Physical abuse, Verbal abuse, Sexual abuse	2011–2012 Behavioral Risk Factor Surveillance System participants *n* = 78,435, 48.6% female, 82.3% Caucasian, adults	Positively associated with cardiovascular disease independent of depression	Salas et al. (14)
Physical abuse, Physical neglect, Emotional abuse, Emotional neglect, Sexual abuse	National Epidemiologic Study of Alcohol and Related Conditions participants *n* = 34,653, 52.1% female, aged ≥20 years	Associated with increased risk of myocardial infarction	Chou and Koenen (15)
Famine exposure	China Cardiometabolic Disease and Cancer Cohort Study participants *n* = 77,925, 69.5% female, aged ≥40 years	Increased risk of diabetes in fetal-exposed individuals	Lu et al. (16)
Famine exposure	REACTION (Risk Evaluation of Cancers in Chinese Diabetic Individuals: A Longitudinal) Study participants *n* = 234,219, 67.9% female, aged ≥40 years	Increased risk of total cardiovascular disease, myocardial infarction, stroke, and coronary heart disease	Du et al. (17)
Physical abuse, Physical neglect, Emotional abuse, Emotional neglect, Sexual abuse	2006–2010 UK Biobank participants *n* = 153,287, 56.4% female, aged 55.9 ± 7.7 years	Increased risk of incident heart failure dose-dependent	Liang et al. (18)
Feel alone, Peer bullied, Relationship with parents, Relationship with neighbors, Relationship with friends, Self-reported health status, Health limitation, Death of parents, Death of siblings, Physical abuse, Parental mental health, Guardians’ bad habits, Hunger, Childhood neighborhood quality, Childhood neighborhood safety	2011–2018 China Health and Retirement Longitudinal Study (CHARLS) participants *n* = 12,981, 51.2% female, aged ≥45 years	Associated with both onset and severity of symptoms and cardiovascular diseases	Liu et al. (19)
Household mental illness, Household substance abuse, Household incarceration, Parental separation/divorce, Witnessing household violence, Physical abuse, Emotional abuse, Sexual abuse	2020 Behavioral Risk Factor Surveillance System participants *n* = 31,242, 54.6% female, 79.8% Caucasian, aged 68.7 ± 7.85 years	Men: household incarceration was most strongly associated with stroke Women: the strongest connection was between physical abuse and stroke, followed by sexual abuse and angina/coronary heart disease	Lee et al. (20)
Material deprivation (family poverty, parental long-term unemployment), Loss or threat of loss in the family (parental and sibling somatic illness and death), Family dynamics (foster care placements, parental and sibling psychiatric illness, parental alcohol and drug abuse, maternal separation)	Danish Life Course Cohort Study participants *n* = 1,263,013, 48.7% female, aged 16–38 years	Higher risk of developing cardiovascular disease in young adulthood	Bengtsson et al. (21)

Certain specific types of ELA have also been associated with cardiovascular events. The risk of hypertension was increased in males who have witnessed violence with a weapon and female victims of violence (12). Fetal exposure to famine increased the risk of diabetes (16), while famine experienced in early life increased the risk of total cardiovascular disease, myocardial infarction, stroke, and coronary heart disease (17). In addition, early exposure to air pollution increased the lifetime burden on the cardiovascular system, contributing to the development of cardiovascular disease (22).

Sex differences are observed in the incidence and effects of ELA. Within the same racial and ethnic population, females are more likely than males to experience ELA (23). The risk of cardiovascular disease increased in women who were exposed to financial difficulties, interpersonal conflicts, and chronic illness in a relative. For men, an increased risk was observed only following chronic illness in a relative (10). Stoke was most strongly associated with physical abuse in women and household incarceration in men (20).

## 3 Possible mechanisms linking ELA and cardiovascular disease in humans

### 3.1 Behavioral factors

Diet, physical activity, nicotine exposure, sleep health, body mass index, blood lipids, blood glucose, and blood pressure are recognized as Life’s Essential 8, the eight most important contributors to cardiovascular health (24). ELA exposure significantly increases the incidence of smoking, alcoholism, physical inactivity, obesity, and poor sleep (Table 2) (1, 38, 40–44).

**Table 2:** Correlation of ELA with cardiovascular risk behaviors.

Type of adversity	Participants	Cardiovascular risk behaviors	References
Household mental illness, Household drinking problem, Household substance abuse, Household member incarceration, Parent separation or divorce, Household physical assault, Victim of physical assault, Victim of verbal abuse, Touched sexually, Forced to touch adult sexually, Forced to have sex	2009 Behavioral Risk Factor Surveillance System participants *n* = 10,277, 51.3% female, aged ≥18 years	Cigarette smoking	Vander Weg (25)
Abuse (physical, verbal), Household dysfunction, Emotional neglect	Taiwan Youth Project participants *n* = 2,903, 50.5% female, aged 22 years	Heavy smoking	Lin and Chiao (26)
Lived with problem drinker/alcoholic, Lived with drug abuser, Parents divorced, Forced sex as a child, Childhood physical abuse by parents, Childhood verbal abuse by parents	2010 Behavioral Risk Factor Surveillance System participants *n* = 19,356, 59.4% female, aged ≥18 years	Physical abuse was associated with smoking in males and females, sexual abuse and verbal abuse were associated with smoking in females but not males	Fuller-Thomson et al. (27)
Physical abuse, Sexual abuse, Verbal abuse, Mental illness in home, Substance abuse in home, Separation/divorce in home, Violence in adults at home, Incarceration at home	2011 Behavioral Risk Factor Surveillance System survey participants *n* = 48,526, 50.43% female, aged ≥18 years	Binge drinking, heavy drinking, smoking	Campbell et al. (28)
Physical abuse, Household alcohol abuse, Household mental illness	US National Longitudinal Survey of Youth 1979 (NLSY79) and NLSY79 Children and Young Adults Survey participants *n* = 2,999 mothers; *n* = 6,596 children	Women are more likely to smoke during pregnancy; their children are more likely to start smoking before age 18	Pear et al. (29)
Household mental health, Household alcohol abuse, Household drug abuse, Household incarceration, Parental separation or divorce, Household physical violence, Child physical abuse, Child verbal abuse, Sexual abuse	2012 Behavioral Risk Factor Surveillance System participants *n* = 39,434, 60.5% female, aged ≥18 years	Heavy and binge drinking in men who lived with a drug abuser as a child; binge drinking in men and women exposed to verbal abuse	Fang et al. (30)
Abuse/child maltreatment, Household dysfunction	Australian Longitudinal Study on Women’s Health participants *n* = 8,915, 100% female, aged 19–26 years	E-cigarette use	Melka et al. (31)
Abuse, Neglect, Household dysfunction	National Epidemiological Survey on Alcohol and Related Conditions-III and National Institute on Alcohol Abuse and Alcoholism participants *n* = 26,855, 41.6% female, aged ≥18 years	High-intensity binge drinking	Jung et al. (32)
Bullying	Meta-analysis of 18 cross-sectional studies *n* = 386,740, 51.8% female, children and adolescents	Physical inactivity and sedentary behavior	García-Hermoso et al. (33)
Childhood maltreatment (emotional, physical, sexual, neglect), Household dysfunction (violence between parents, parental separation, household substance abuse, mental illness, incarceration)	Meta-analysis of 10 observational studies *n* = 118,691	46% increase in the risk of adult obesity following exposure to multiple adversities	Wiss and Brewerton (34)
Abuse (emotional, physical, and sexual), Household stressors	Community Health Needs Assessment in Florida participants *n* = 14,056, 74.1% female, aged ≥18 years	Cigarette and e-cigarette use	Martinasek et al. (35)
Child in care, Physical neglect, Offenders, Parental separation, Mental illness, Alcohol abuse	1958 National Child Development Study participants *n* = 7,414, 51.5% female, aged 7–42 years	Smoking by age 16 and long-term smoking	Joannès et al. (36)
Childhood abuse (emotional, physical, sexual), Household dysfunction (mental illness, substance use, incarceration, separation/divorce, witnessing domestic violence)	2019 Behavioral Risk Factor Surveillance System survey participants *n* = 41,322, 50.7% female, aged 18–64 years	Binge drinking risk was 1.36 times higher in females who experienced two types of adversities, and 1.58 times higher for females who experienced three	Baiden et al. (37)
Sexual abuse, Physical abuse, Emotional abuse, Neglect, Family dysfunction	Meta-analysis of 9 articles *n* = 108,330, participants from 5 high-income countries	Sleep deficiency and poor sleep quality	Yu et al. (38)
Physical abuse, Sexual abuse, Verbal abuse, Mental illness, Substance abuse, Separation/divorce, Violence in adults, Incarceration	2020 Behavioral Risk Factor Database participants *n* = 42,786, 55.8% female, aged ≥65 years	Short and long sleep duration	Cheng et al. (39)

ELA increases the risk of both cigarette and e-cigarette smoking (25, 26, 31, 35), with exposed individuals more likely to start smoking by 16 years of age and smoke for a lifetime (36). Physical abuse was associated with smoking in both males and females; sexual and verbal abuse were associated with smoking in females but not males (27). This correlation has intergenerational effects, maternal exposure to childhood physical abuse was associated with an increased risk of both smoking during pregnancy and the child smoking (29).

ELA is also associated with high-intensity binge drinking (32). Heavy and binge drinking was more common in men who lived with a drug abuser as a child than in those who did not, and verbal abuse was correlated with binge drinking in both men and women (30). The greater the total number of adversities experienced, the higher the probability of binge drinking (28, 37). Although some studies suggest that moderate drinking may be beneficial for cardiovascular health, large cohort studies have shown that all levels of alcohol consumption are associated with an increased cardiovascular risk (45–47).

A meta-analysis showed that physical inactivity increased among children and adolescents exposed to bullying (33). A separate meta-analysis of 10 observational studies showed that the risk of obesity in adulthood increased 46% following ELA (34). Physical inactivity and obesity have been closely associated with high blood lipid, glucose, and blood pressure levels, which are major contributors to cardiovascular disease (48, 49).

A systematic review and meta-analysis indicated that ELA has a considerable impact on long-term sleep health: the greater the exposure, the higher the risk of sleep disorders (38). ELA exposure was associated with both short and long sleep duration: victims of childhood sexual abuse were 146% more likely to short sleep and 99% more likely to long sleep; reporting more than four types of adversities raised these risks to 3.10 and 2.13 times, respectively (39). Both short and long sleep duration and poor sleep quality are associated with an increased risk of all-cause mortality and cardiovascular events (50, 51).

### 3.2 Mental factors

ELA is associated with the development of mental illnesses in adulthood, including anxiety, depression, post-traumatic stress disorder (PTSD), and schizophrenia. Cohort studies have shown that ELA increases the incidence of anxiety, depression, and PTSD (52–54). ELA exposure has been associated with both the onset and severity of depression and cardiovascular disease (19). The number of adversity types experienced was a nonlinear predictor of depression (55). A meta-analysis including over 200,000 participants showed that individuals maltreated in childhood had an increased risk of depression and cardiometabolic disease, and were three times more likely to have comorbid depression and cardiometabolic disease (56). A systematic review and meta-analysis associated childhood trauma with the severity of hallucinations and delusions in individuals with psychotic disorders (57). In addition, patients with schizophrenia report more exposure to ELA than healthy controls (58).

Mental illness increases the burden on the cardiovascular system through biological pathways such as elevated cortisol, oxidative stress, inflammation, and autonomic nervous system dysfunction, as reviewed in detail by Treur et al. (59). Patients with severe mental illness have twice the cardiovascular mortality rate of the general population (60, 61). Both anxiety and depression are associated with an increased risk of mortality owing to cardiovascular events (62, 63). Schizophrenia reduces life expectancy by 15–20 years, with 40–50% of deaths due to cardiovascular disease (64). Thus, ELA may predispose individuals to cardiovascular diseases later in life through their susceptibility to mental illnesses.

### 3.3 Biological pathways

ELA disturbs the neuroendocrine and immune systems in adulthood; this dysregulation has been associated with cardiovascular diseases. Hypothalamic–pituitary–adrenal (HPA) axis dysfunction has been observed in individuals exposed to ELA (65–68). A meta-analysis indicated that cortisol levels affected by ELA (67). Bullying, emotional abuse, and cumulative exposure to adversity have been associated with low daytime salivary cortisol levels in adolescents (66). Increased levels of hair cortisone were detected among middle-aged and older individuals exposed to ELA (65, 68). Meta analyses have indicated that ELA increases levels of inflammatory mediators such as C-reactive protein, interleukin-6, and tumor necrosis factor-α (69, 70). These effects persist into late adulthood (71). Dysregulated inflammation and cortisol levels may contribute to the development of mental illnesses such as depression (72–74), increasing the risk of cardiovascular disease.

ELA has been reported to directly increase circulating endothelin-1 levels in young adulthood, with greater numbers of adversity types faced further increasing endothelin-1 levels (75). Endothelin-1 is a peptide produced by the vascular endothelium in response to stress (76, 77) and is implicated in the pathogenesis of cardiovascular diseases (78). Physical exercise decreases endothelin-1 levels among women exposed to ELA and improves cardiovascular psychophysiological outcomes; endothelin-1 may therefore be a potential therapeutic target for the treatment of cardiovascular diseases following ELA (79).

## 4 Impact of ELA on the cardiovascular system in rodents

### 4.1 Rodent models of ELA

In humans, ELA primarily involves abuse, neglect, and household dysfunction. To mimic these adversities, several types of rodent models have been developed: (1) Maternal separation, in which pups are removed from their mother for several hours per day (at different times each day) during the postnatal period (2). Social deprivation, in which pups are separated from both their mother and littermates for several hours per day (at different times each day) during the postnatal period (3). Limited bedding and nesting, in which the mother and pups have limited access to nesting material during the postnatal period (4). Early weaning, in which pups are weaned before postnatal day 21, usually on postnatal day 17. Of these, maternal separation is the most widely used. To simulate multiple ELA exposures, these models are used in combination, such as maternal separation combined with limited bedding and nesting and maternal separation combined with early weaning (80, 81). These rodent models effectively simulate the negative outcomes of ELA, behavioral experiments have shown that rodents exposed to ELA elicit negative psychopathological outcomes in adulthood. Mice exposed to maternal separation and limited nesting were more susceptible to depression-like behaviors after social defeat (82). Social deprivation during postnatal days 1–14 increased the risk of social dysfunction later in life (83).

### 4.2 Mechanisms underlying the effect of ELA on the cardiovascular system in rodents

Consistent with results from human studies, ELA in rodents negatively affects the cardiovascular system (Table 3). Neuroendocrine, immune system and vascular dysfunction have been identified following ELA in rodents. Dysfunction in the HPA axis and renin-angiotensin-aldosterone system were detected in rats and mice following maternal separation, as evidenced by increased levels of corticotropin-releasing hormone, adrenocorticotropic hormone, corticosterone, and angiotensin II (84, 86, 92). Circulating endothelin-1 levels were significantly increased in rats following maternal separation (85). In a mouse model of maternal separation, acute mixing stress induced lower blood pressure and heart rate responses (87). Maternal separation in rats resulted in misprogramming of resistance artery smooth muscle, increased vasoconstriction and blood pressure (88), and blood–brain barrier dysfunction (84, 95). ELA also increased oxidative stress and inflammation. Levels of the reactive oxygen species nicotinamide adenine dinucleotide phosphate oxidases 2 and 4 (89, 90) and the inflammatory protein interleukin-1β and Toll-link receptor 4 (93, 95) were increased in rodents following ELA. ELA may be linked to oxytocin signaling, cardiac oxytocin receptors decreased following maternal separation on postnatal days 1–21, but increased following maternal separation on postnatal days 12–16 (91). Maternal separation and early weaning increased adiposity in female mice, which may be the mechanism underlying the development of obesity and diabetes following ELA (94).

**Table 3:** Molecular and/or structural alteration following ELA on cardiovascular system in rodent.

Animal	Model	Molecular and/or structural alteration	References
Wistar rats	Prenatal stress (E10-20) and maternal separation (P2-20)	Plasma corticosterone, blood–brain barrier functional development	Gomez-Gonzalez and Escobar (84)
Rats	Maternal separation (P2-14)	Circulating endothelin-1, endothelin A and B receptors in aortic tissue	Loria et al. (85)
Wistar Kyoto rats	Maternal separation (P2-14)	Angiotensin II	Loria et al. (86)
BALB/c mice	Maternal separation (P2-14)	Blood pressure and heart rate, induced by acute mixing stress	Pote et al. (87)
Sprague–Dawley rats	Maternal separation (P2-14)	Small resistance mesenteric arteries	Reho and Fisher (88)
C57BL/6 J mice	Maternal separation (P2-16) with early weaning at P17	Nicotinamide adenine dinucleotide phosphate oxidase 2 and 4	Ho et al. (89)
Male Albino Wistar rats	Maternal separation (P2-14)	Reactive oxygen species, mitochondrial glutathione, ATP, cytochrome c	Sahafi et al. (90)
C57BL/6 mice	Long-Term Separation Stress: maternal separation (P1-21) Short-Term Separation Stress: maternal separation (P14-16)	Oxytocin receptor	Wigger et al. (91)
CD-1 mice	Maternal separation (P1-21)	Corticotropin-releasing hormone, adrenocorticotropic hormone, corticosterone	Campana et al. (92)
NMRI mice	Maternal separation (P2-14)	Malondialdehyde, nitrite, interleukin-1β, Toll-like receptor 4	Arabi et al. (93)
C57BL/6 J female mice	Maternal separation (P2-16) with early weaning at P17	Adipocyte, aldosterone, corticosterone	Leachman et al. (94)
Wistar rats	Maternal separation (P1-14) and lipopolysaccharide injection in juvenility or adulthood	Serum proinflammatory cytokines, blood–brain barrier permeability	Solarz et al. (95)

## 5 Discussion and future directions

Despite the progress in community and family support, ELA remains highly prevalent and contributes to the morbidity and mortality of cardiovascular diseases worldwide. The prevalence of ELA varies between countries and regions, and between sexes. Over time, the prevalence of different subtypes of ELA changes. This means that the adoption of rigorous sampling strategies is required to dynamically surveil the prevalence of subtypes of ELA and minimize data bias (7). It also means that policymakers need to be flexible in the use of prevention strategies to reduce the incidence of ELA. Numerous cohort studies have revealed the association between ELA and cardiovascular disease, with the risk increasing with greater ELA exposure. However, the frequency, intensity, exposure duration, and exposure timing of each subtype of ELA, which may also have an impact on cardiovascular outcomes, have not been fully assessed (96). Each individual’s childhood experience is unique and may experience some specific subtypes of ELA. Therefore, how to accurately evaluate the effect of ELA on an individual’s cardiovascular system requires further investigation.

Mechanistic studies in humans have shown that ELA increases cardiovascular health risk behaviors, susceptibility to mental illnesses, and neuroendocrine and immune system dysfunction. But whether ELA directly affects the development of cardiovascular disease or whether the association between ELA and cardiovascular disease is fully mediated through risk behaviors remains to be determined. Rodent models have been developed to study the mechanisms underlying the effects of ELA on cardiovascular outcomes and result in cardiovascular dysfunction similar to that observed in humans. Rodents exposed to ELA showed similar behavioral tendencies to humans exposed to ELA, such as alcohol consumption (97), drug addiction (98), and depression-like behavior (82). However, whether these behavioral effects contribute to cardiovascular outcomes remains largely unknown. Rodent models provide an important research tool as they allow the association between ELA and cardiovascular disease to be analyzed in a homogenous population under controlled conditions. However, these models cannot fully replicate all of ELA subtypes. Therefore, combining cohort and rodent studies to analyze the association between ELA and cardiovascular diseases may be a feasible future research strategy.

## Author contributions

HT: Conceptualization, Resources, Visualization, Writing – original draft, Writing – review & editing. HZ: Writing – review & editing. JC: Writing – review & editing. HR: Writing – review & editing. YG: Funding acquisition, Project administration, Supervision, Writing – review & editing. XJ: Conceptualization, Funding acquisition, Project administration, Resources, Supervision, Writing – review & editing.
